# Trends in the Stroke Death Rate Among Mississippi Adults, 2000–2016

**DOI:** 10.5888/pcd16.180425

**Published:** 2019-02-14

**Authors:** Vincent L. Mendy, Rodolfo Vargas, Marinelle Payton, Jennifer N. Sims, Lei Zhang

**Affiliations:** 1 Department of Epidemiology and Biostatistics, School of Public Health, Jackson State University, Jackson, Mississippi; 2 Office of Health Data and Research, Mississippi State Department of Health, Jackson, Mississippi; 3 Center of Excellence in Minority Health and Health Disparities, Institute of Epidemiology and Health Services Research, School of Public Health, Jackson State University, Jackson, Mississippi; 4 Department of Behavioral and Environmental Health, School of Public Health, Jackson State University, Jackson, Mississippi

## Abstract

**Introduction:**

On average, more than 1,700 people in Mississippi die from stroke annually, but data on trends by age, sex, and race in Mississippi are limited. We examined trends in the stroke death rate among adults in Mississippi aged 35 or older by age, sex, and race.

**Methods:**

We used Mississippi Vital Statistics data to calculate age-specific death rates for stroke among people in Mississippi aged 35 or older from 2000 to 2016. We identified cases according to underlying cause-of-death codes from the *International Classification of Diseases, Tenth Revision *(ICD-10). We used Joinpoint software to calculate annual percentage change (APC) and the average annual percentage change (AAPC) in death rates for stroke by age, sex, and race (non-Hispanic black and non-Hispanic white).

**Results:**

Among adults aged 35 or older, the age-adjusted stroke death rate declined 30.7% from 141.3 per 100,000 population in 2000 to 97.9 per 100,000 population in 2016, with an AAPC of −2.4% (95% confidence interval, −3.1% to −1.6%). Stroke death rates declined significantly among both men and women in the first trend segment (2000–2009 for men and 2000–2007 for women) but did not decline in the second trend segment (2009–2016 for men and 2007–2016 for women). Non-Hispanic black men had the smallest decline in stroke death rates during the full study period. Among people aged 55 to 64 and non-Hispanic white men, rates shifted from a significant annual decline during the first segment to a significant annual increase during the second segment.

**Conclusion:**

Age-adjusted stroke death rates among adults in Mississippi aged 35 or older declined significantly between 2000 and 2016, but trends differed by age, race, and sex. Clinical and community interventions aimed at reducing stroke risk factors, particularly for adults aged 55 to 64, are needed in Mississippi.

**Summary**

**What is already known on this topic?**

In 2016, stroke was the sixth leading cause of death in Mississippi.

**What is added by this report?**

Data from the Mississippi Vital Statistics indicated that among Mississippians aged 35 or older, the age-adjusted stroke death rate declined by 30.7% from 2000 to 2016 with an average annual percentage change of of −2.4%; however, the magnitude and timing of the decline differed by age group, race, and sex. Among people aged 55 to 64 and non-Hispanic white men, rates shifted from a significant annual decline during the first segment to a significant annual increase during the second segment.

**What are the implications for public health practice?**

Clinical and community interventions aimed at reducing stroke risk factors, particularly for adults aged 55 to 64, are needed in Mississippi.

## Introduction

On average, more than 1,700 people in Mississippi die from stroke each year (1). In 2016, stroke was the sixth leading cause of death in Mississippi, accounting for 5.4% of all deaths (1). Furthermore, the 2016 stroke death rate in Mississippi was 1.4 times greater than the national rate (2) and disproportionately affected black people and men (1). In 2000, Mississippi had the sixth highest age-adjusted stoke death rate in the United States, and in 2016, the second highest rate (2). Epidemiological studies demonstrate a decline in stroke death rates in the United States in recent decades (3,4), but this decline varies by geography and race/ethnicity (5,6). A recent study using data from 2000 to 2015 found that after decades of decline, stroke death rates in the United States began to decline more slowly, stall, or reverse among certain subpopulations (7). For example, from 2013 to 2015, the stroke death rate increased in the South Census Region, which includes Mississippi (7), a state in the Stroke Belt (8) (an 8-state region with disproportionately high stroke mortality rate in the southeastern United States) that perennially has some of highest cardiovascular-related deaths in the country (2).

Assessing trends in stroke deaths by race and sex would provide important information for community and population health program managers, policy makers, advocacy organizations, and health professionals as they seek to implement stroke awareness, prevention, and treatment programs that address stroke disparities in Mississippi. Few studies have examined annual changes in stroke death rates in Mississippi. To address this gap, we calculated the annual percentage change (APC, trend segment) and the average annual percentage change (AAPC) in age-adjusted stroke death rates among Mississippi adults aged 35 or older from 2000 to 2016. In addition, we examined differences in the AAPC by age, sex, and race.

## Methods

We extracted data on the number of deaths due to stroke among adults aged 35 or older for each year from 2000 to 2016 from Mississippi Vital Statistics (1). In 2016, nearly all (99.2%) stroke deaths in Mississippi occurred among people aged 35 or older. We used underlying cause-of-death codes from the *International Classification of Diseases, Tenth Revision* (ICD-10) to identify stroke deaths; we included all cases with codes I60–I69 (1). We then used Mississippi population census counts to calculate age-adjusted stroke death rates and standard errors for the overall population, by age group (35–54, 55–64, 65–74, 75–84, and ≥85), race (non-Hispanic black or non-Hispanic white), sex (male or female), and race and sex (non-Hispanic black male and female or non-Hispanic white male and female) using SAS version 9.4 (SAS Institute Inc). We adjusted for age by using the direct method and the 2000 US standard projected population (9).

We then exported age-adjusted stroke death rates and standard errors to the US Surveillance, Epidemiology, and End Results (SEER) Joinpoint software (version 4.6.0.0) (https://surveillance.cancer.gov/joinpoint/) to calculate the AAPC in stroke death rates for the overall Mississippi population as well as by race, by sex, and by race and sex. We restricted analyses to non-Hispanic black and non-Hispanic white Mississippians; these racial groups accounted for 97.7% of the Mississippi population in the 2000 Census and 97.0% of the state’s population in 2016 (1). Joinpoint regression analysis identifies trend breaks (joinpoints) or points of significant change in a trend. This analysis identified periods with distinct log-linear trends in stroke death rates (10). Using the Bayesian information criterion to select the most parsimonious model with the best fit, we specified a maximum of 3 joinpoints (7,10). We used the slopes of the models to calculate the APC for each trend segment and the AAPC (the weighted average of the APCs). For each AAPC, we calculated 95% confidence intervals (CIs) and used a *P* value of .05 to determine significant differences. Our investigation was approved by the Jackson State University Institutional Review Board.

## Results

From 2000 to 2016 in Mississippi, 27,526 stroke deaths occurred among adults aged 35 or older. Among this population, the overall age-adjusted stroke death rate declined by 30.7% from 2000 (141.3 per 100,000 population) to 2016 (97.9 per 100,000 population) with an AAPC of −2.4%, (95% CI, −3.1% to −1.6%) (Table). However, this period consisted of 2 distinct segments: the overall rate first declined significantly by 5.0% (95% CI, −6.1% to −3.9%) per year from 2000 to 2008 and then increased nonsignificantly by 0.4% (95% CI, −0.8% to 1.6%) per year from 2008 to 2016.

**Table:** Trends in Age-Adjusted Stroke Death Rates Among Mississippi Adults Aged ≥35, 2000–2016^a^

Characteristic	No. of Stroke Deaths (Age-Adjusted Rate)^b^		AAPC (95% CI)^c^	Trend Segment 1		Trend Segment 2	
	2000	2016	2000–2016	Years	APC^d^ (95% CI)	Years	APC (95% CI)
**Overall**	1,947 (141.3)	1,681 (97.9)	−2.4^e^ (−3.1 to −1.6)	2000–2008	−5.0^e^ (−6.1 to −3.9)	2008–2016	0.4 (−0.8 to 1.6)
**Sex**							
Male	741 (142.1)	758 (106.3)	–2.2^e^ (−3.3 to −1.2)	2000–2009	−5.0^e^ (−6.3 to −3.7)	2009–2016	1.5 (−0.6 to 3.6)
Female	1,206 (137.6)	923 (90.6)	−2.5^e^ (−3.4 to −1.7)	2000–2007	−5.5^e^ (−6.9 to 4.0)	2007–2016	−0.2 (−1.3 to 1.0)
**Age group, y**							
35–54	169 (21.5)	122 (16.4)	−1.1 (−2.2 to 0.1)	2000–2016	−1.1 (−2.2 to 0.1)	—f	—f
55–64	177 (72.0)	256 (67.2)	−0.4 (−2.1 to 1.3)	2000–2012	−3.5^e^ (−4.8 to −2.2)	2012–2016	9.5^e^ (2.6 to 16.8)
65–74	304 (163.7)	315 (118.4)	−2.8^e^ (−3.6 to −2.0)	2000–2016	−2.8^e^ (−3.6 to −2.0)	—f	—f
75–84	646 (562.1)	470 (350.9)	−2.6^e^ (−3.5 to −1.6)	2000–2009	−4.9^e^ (−6.1 to −3.7)	2009–2016	0.5 (−1.44 to 2.55)
≥85	651 (1,517.8)	518 (1,016.4)	−2.7^e^ (−4.8 to −1.2)	2000–2007	−7.0^e^ (−9.5 to −4.4)	2007–2016	0.9 (−1.1 to 2.87)
**Race^g^**							
Black	628 (168.5)	585 (118.4)	−2.4^e^ (−3.1 to −1.8)	2000–2016	−2.4^e^ (−3.1 to −1.8)	—f	—f
White	1,319 (132.8)	1,096 (89.5)	−2.4^e^ (−3.1 to −1.6)	2000–2008	−5.7^e^ (−6.8 to −4.6)	2008–2016	1.1 (−0.2 to 2.3)
**Race^g^ and sex**							
Black female	376 (161.5)	299 (102.6)	−3.0^e^ (−3.7 to −2.3)	2000–2016	−3.0^e^ (−3.7 to −2.3)	—f	—f
White female	830 (129.0)	624 (85.2)	−2.4^e^ (−3.3 to −1.4)	2000–2007	−5.8^e^ (−7.5 to −4.1)	2007–2016	0.4 (−0.9 to 1.8)
Black male	252 (175.3)	286 (140.8)	−2.1^e^ (−3.0 to −1.2)	2000–2016	−2.1^e^ (−3.0 to −1.2)	—f	—f
White male	489 (134.0)	472 (93.5)	−2.5^e^ (−3.7 to −1.4)	2000–2009	−6.1^e^ (−7.5 to −4.7)	2009–2016	2.3^e^ (0.1 to 4.6)

Abbreviations: AAPC, average annual percentage change; APC, annual percentage change; CI, confidence interval.

a Data source: Mississippi Vital Statistics (1).

b Per 100,000 population, adjusted to the 2000 US standard population with age groups 35–54, 55–64, 65–74, 75–84 and ≥85 years.

c The AAPC is a weighted average of the APCs calculated by joinpoint regression.

d The APC is based on age-adjusted rates to the 2000 US standard population.

e Significant at *P* < .05.

f Dashes indicate that the best-fit joint model did not include that trend segment.

g Analyses were restricted to non-Hispanic white and non-Hispanic black.

Among men, the age-adjusted stroke death rate declined by 25.2% from 2000 (142.1 per 100,000 population) to 2016 (106.3 per 100,000 population). The overall AAPC for this group was −2.2% (95% CI, −3.3% to −1.2%) from 2000 to 2016; however, this period consisted of 2 distinct segments: a significant APC of −5.0% (95% CI, −6.3% to −3.7%) from 2000 to 2009 and a nonsignificant APC of 1.5% (95% CI, −0.6% to 3.6%) from 2009 to 2016. Among women, the age-adjusted stroke death rate declined by 34.2% from 2000 (137.6 per 100,000 population) to 2016 (90.6 per 100,000 population), with a significant AAPC of −2.5% (95% CI, −3.4% to −1.7%) from 2000 to 2016. As it did among men, the trend changed over time among women, with a significant APC of −5.5% (95% CI, −6.9% to 4.0%) from 2000 to 2007 and a nonsignificant APC of −0.2% (95% CI, −1.3% to 1.0%) from 2007 to 2016.

The stroke death rate declined among all age groups from 2000 to 2016; however, the decline was not significant among adults aged 35 to 54 or adults aged 55 to 64. The decline was significant among adults aged 65 to 74 (AAPC, −2.8%, 95% CI, −3.6% to −2.0%), adults aged 75 to 84 (AAPC, −2.6%, 95% CI, −3.5% to −1.6%), and adults aged 85 or older (AAPC, −2.7%, 95% CI, −4.8% to −1.2%). In addition, the trend in the stroke death rate changed during the entire study period for certain age groups. Among adults aged 55 to 64, the stroke death rate declined significantly by 3.5% per year from 2000 to 2012 and then increased significantly by 9.5% per year from 2012 to 2016. Among adults aged 75 to 84, rates declined significantly by 4.9% per year from 2000 to 2009 and then increased nonsignificantly by 0.5% per year from 2009 to 2016. Among adults aged 85 or older, the rate declined significantly by 7.0% per year from 2000 to 2007 and then increased nonsignificantly by 0.9% per year from 2007 to 2016.

Among non-Hispanic black adults, the age-adjusted stroke death rate declined by 29.7% from 2000 (168.5 per 100,000 population) to 2016 (118.4 per 100,000 population), with a significant AAPC of −2.4% (95% CI, −3.1% to −1.8%) from 2000 to 2016. Among non-Hispanic white adults, the age-adjusted stroke death rate declined by 32.6% from 2000 (132.8 per 100,000 population) to 2016 (89.5 per 100,000 population), with a −2.4% (95% CI, −3.1% to −1.6%) AAPC in this period. The trend among non-Hispanic white adults was in 2 distinct segments: rates declined significantly by 5.7% per year from 2000 to 2008 and then increased nonsignificantly by 1.1% per year from 2008 to 2016.

Among non-Hispanic black women, the age-adjusted stroke death rate declined by 36.5% from 2000 (161.5 per 100,000 population) to 2016 (102.6 per 100,000 population), with a significant AAPC of −3.0% (95% CI, −3.7% to −2.3%) during this period. Among non-Hispanic white women, the age-adjusted stroke death rate declined by 34.0% from 2000 (129.0 per 100,000 population) to 2016 (85.2 per 100,000 population), with a significant AAPC of −2.4% (95% CI, −3.3% to −1.4%). The rate declined significantly by 5.8% per year from 2000 to 2007 and then increased nonsignificantly by 0.4% per year from 2007 to 2016. Among non-Hispanic black men, the age-adjusted stroke death rate declined by 19.7% from 2000 (175.3 per 100,000 population) to 2016 (140.8 per 100,000 population), with a significant AAPC of −2.1% (95% CI, −3.0% to −1.2%) during this period. For non-Hispanic white men, the age-adjusted stroke death rate declined by 30.2% from 2000 (134.0 per 100,000 population) to 2016 (93.5 per 100,000 population), with a significant AAPC of −2.5% (95% CI, −3.7% to −1.4%) from 2000 to 2016. Again, the trend was in 2 distinct segments: rates declined significantly by 6.1% per year from 2000 to 2009 and then increased significantly by 2.3% per year from 2009 to 2016 (Table and Figure).

**Figure:**
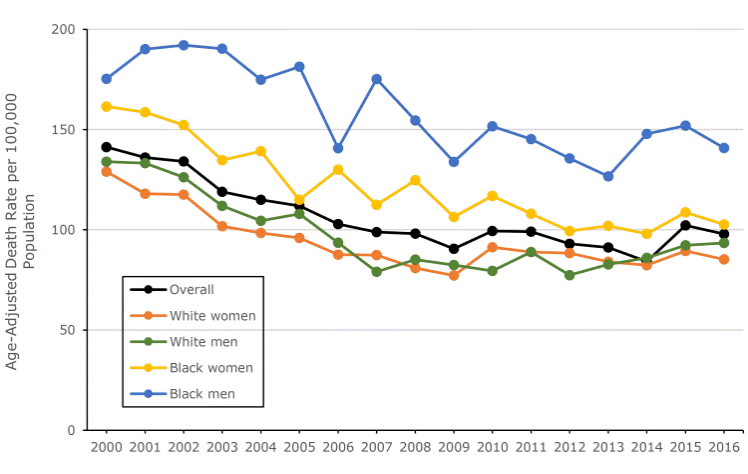
Trends in the age-adjusted stroke death rate among Mississippi adults aged 35 or older by race and sex, 2000 through 2016. Analyses were restricted to non-Hispanic white and non-Hispanic black.

**Table:** 

Year	Overall	White Women	White Men	Black Women	Black Men
2000	141.3	129.0	134.0	161.5	175.3
2001	136.1	118.0	133.2	158.7	190.1
2002	134.1	117.6	126.2	152.3	192.1
2003	119.0	101.8	112.0	134.8	190.3
2004	115.0	98.4	104.5	139.2	174.9
2005	112.0	95.9	108.0	115.0	181.4
2006	102.9	87.6	93.6	130.0	140.7
2007	98.9	87.4	79.1	112.5	175.2
2008	98.1	80.9	85.1	124.7	154.6
2009	90.5	77.2	82.4	106.4	133.9
2010	99.4	91.3	79.5	116.9	151.6
2011	99.1	88.9	88.9	108.1	145.3
2012	93.0	88.4	77.4	99.4	135.6
2013	91.2	84.0	82.7	102.0	126.7
2014	84.2	82.3	86.0	98.0	147.8
2015	102.2	89.5	92.3	108.7	152.0
2016	97.9	85.2	93.5	102.6	140.8

## Discussion

In Mississippi, among adults age 35 or older, the age-adjusted stroke death rate declined by 30.7% between 2000 and 2016; however, the magnitude and timing of the decline differed by age group, race, and sex. The overall age-adjusted stroke death rate declined significantly by 5% per year during the first trend segment (2000 to 2008), but did not decline in the second trend segment (2008 to 2016). Our finding of declining age-adjusted stroke death rates is consistent with findings on national trends reported in 2017 (7), although the national annual decline in the magnitude of the age-adjusted stroke death rate (3.1%) was larger than the decline we observed in Mississippi (2.4%). Additionally, a previous study found that age-adjusted stroke death rates declined in the Stroke Belt, which includes Mississippi (11).

Researchers have documented several reasons for the decline in stroke death rates, including reduced incidence and case fatality rates and improved survival after stroke (12). For example, data from the Atherosclerosis Risk in Communities Study (ARIC) , which included participants from Mississippi, showed a significant decline in stroke incidence from 1987 to 2011 among both white and black participants and among both men and women (3). Furthermore, improved control of risk factors such as high blood pressure and high cholesterol have contributed to a decline in stroke incidence, which may have contributed to lower stroke death rates (3,12–14). Data reported in 2008 from the Jackson Heart Study, which includes the counties of Hinds, Madison, and Rankin in Mississippi, showed that of the 5,249 adult participants, two-thirds (66.4%) had their blood pressure under control (15).

Age-adjusted stroke death rates declined by one-quarter among men and by one-third among women during our full study period; for each sex, the magnitude of decline during the full study period was similar to the decline during the first trend segment. ARIC showed a similar decline in stroke incidence among men and women (3). We found no decline in age-adjusted stroke death rates for either men or women in the second trend segment. Increases in obesity and diabetes prevalence may explain the lack of a decline in stroke death rates (16,17).

Our finding that the magnitude of the decline in the stroke death rate increased with age is similar to the results reported in 2011 by Howard and colleagues in the Reasons for Geographic and Racial Differences in Stroke (REGARDS) study, which includes Mississippi (18). In our study, the stroke death rate reversed significantly among adults aged 55 to 64: after declining by 3.5% per year during the first trend segment (2000–2012), the rate increased by 9.5% per year during the second trend segment (2012–2016). During the full period, the magnitude of decline in the age-adjusted stroke death rate among black and white participants was similar. However, the trend in the age-adjusted stroke death rate changed over time among white participants but not black participants. White participants had a significant decline during the first trend segment but no significant change during the second segment.

The reasons for the reversal in the stroke death rate are not well understood (7), although researchers have attributed a slowing decline in the stroke death rate to increases in obesity, diabetes, and poor diet in the past few decades (7). In 2015, we reported significant increases in the prevalence of obesity (body mass index [BMI] ≥30 kg/m^2^, APC, 2.9%) and extreme obesity (BMI ≥40 kg/m^2^, APC, 3.6%) among men, women, black participants, and white participants in Mississippi from 2001 to 2010 (19). In addition, we found significant increases in the prevalence of high cholesterol (APC, 4.22%), obesity (APC, 3.65%), and diabetes (APC, 3.54%) in the 18-county Mississippi Delta Region from 2001 to 2010 (20). The observed racial and sex disparities in age-adjusted stroke death rates may be due to the differences in the prevalence and trends of stroke risk factors such as obesity, diabetes, and high cholesterol among Mississippi adults (19,20). In addition, a report in 2012 showed an increased incidence of ischemic stroke among both black and white adults aged 20 to 54 (21). The reversal of the decline in the stroke death rate among adults aged 55 to 64 and white men is concerning given the current burden of stroke in Mississippi — in 2016, stroke was the sixth leading cause of death in the state. Thus, the reasons for the reversal in the stroke death rate in these groups warrant further investigation.

Our study has several limitations. First, reliance on death certificates may introduce bias because of the misclassification of the primary cause of death (12,22). Second, research found that the sensitivity of nosologist-coded stroke with physician adjudication was 68%, whereas the specificity was 95%, which could lead to the under-reporting of stroke deaths (12). The major strength of the study was the use of the most recent statewide stroke death rates (from the past 17 years), all of which were calculated by using the same methods.

From 2000 to 2016, the overall age-adjusted stroke death rate declined significantly among adults in Mississippi aged 35 or older, but the magnitude and timing of this decline varied by age, race, and sex. Among adults aged 55 to 64 and non-Hispanic white men, the stroke death rate increased significantly during the second trend segment (2012–2016 and 2009–2016, respectively). Targeted clinical and community interventions that address stroke risk factors (23), particularly for adults aged 55 to 64, are needed in Mississippi. These interventions may help reduce the burden of stroke and associated disparities and produce future reductions in stroke death rates in Mississippi. National, state, and local collaborative efforts are addressing stroke risk factors in the state. These efforts include those of the Mississippi Delta Health Collaborative, which is implementing programs in the 18-county Mississippi Delta region that aim to prevent and reduce heart disease, stroke, and associated risk factors, with a focus on the ABCS (aspirin for those at risk, blood pressure control, cholesterol management, and smoking cessation) of heart disease and stroke prevention (www.healthyms.com/MDHC).
